# Modeling Alzheimer’s disease related phenotypes in the Ts65Dn mouse: impact of age on Aβ, Tau, pTau, NfL, and behavior

**DOI:** 10.3389/fnins.2023.1202208

**Published:** 2023-06-28

**Authors:** Cassia Overk, Emma Fiorini, Chiara Babolin, Marija Vukicevic, Catherine Morici, Rime Madani, Valerie Eligert, Marie Kosco-Vilbois, Amanda Roberts, Ann Becker, Andrea Pfeifer, William C. Mobley

**Affiliations:** ^1^Department of Neurosciences, University of California, San Diego, La Jolla, CA, United States; ^2^AC Immune SA, Lausanne, Switzerland; ^3^Animal Models Core Facility, The Scripps Research Institute, La Jolla, CA, United States

**Keywords:** DS-AD, animal model, age, Down syndrome, Alzheimer’s disease, tau, Abeta, behavior

## Abstract

**Introduction:**

People with DS are highly predisposed to Alzheimer’s disease (AD) and demonstrate very similar clinical and pathological features. Ts65Dn mice are widely used and serve as the best-characterized animal model of DS.

**Methods:**

We undertook studies to characterize age-related changes for AD-relevant markers linked to Aβ, Tau, and phospho-Tau, axonal structure, inflammation, and behavior.

**Results:**

We found age related changes in both Ts65Dn and 2N mice. Relative to 2N mice, Ts65Dn mice showed consistent increases in Aβ40, insoluble phospho-Tau, and neurofilament light protein. These changes were correlated with deficits in learning and memory.

**Discussion:**

These data have implications for planning future experiments aimed at preventing disease-related phenotypes and biomarkers. Interventions should be planned to address specific manifestations using treatments and treatment durations adequate to engage targets to prevent the emergence of phenotypes.

## 1. Introduction

Down syndrome (DS), or trisomy 21, affects one in 650–1,000 newborns globally (CDC, n.d.). Those with DS are highly predisposed to Alzheimer’s disease (AD), a condition referred to as DS with AD (DS-AD). With increased longevity, the prevalence of this condition has increased. Indeed, due to advances in medical care, the average life expectancy for those with DS has increased from ~5 years in the 1950s to ~54 years today (Yang et al., 2002; Presson et al., 2013; Iulita et al., 2022). Consistent with this finding, the primary cause of death of those with DS is now DS-AD (Strydom et al., 2013; Iulita et al., 2022).

AD and DS-AD are marked by similar clinical and pathological features. The onset of dementia in DS features changes in behavior, memory, language, and executive function (reviewed in Overk and Mobley, 2022). The classical neuropathological features of AD, including mature neuritic plaques and neurofibrillary tangles, are present in essentially all those with DS by age 40 (Wisniewski et al., 1985). In addition, AD clinical biomarkers demonstrate very similar patterns for regional distribution and evolution in DS (Rafii et al., 2021). Biomarkers specific for AD include decreases in cerebral spinal cord fluid (CSF) and plasma Aβ42 and the Aβ42/Aβ40 ratio and increases in CSF and plasma phospho-Tau (pTau) species. Those with DS whose age puts them at increased risk show the same changes in CSF amyloid β (Aβ) species and CSF and plasma pTau. Though not specific to AD, DS-AD also demonstrates evidence of axonal injury through age-related increases in neurofilament light protein (NFL) and markers of inflammation. The median age for diagnosis of dementia in DS is ~54 years; approximately 60% have dementia by age ~ 60 and after age 65 more than 80% are demented (Hartley et al., 2015; McCarron et al., 2017). The concordance of clinical and neuropathological findings raises the possibility of common pathogenetic mechanisms underlying AD and DS-AD and the view that elucidating pathogenesis in those with DS may inform AD pathogenesis. Though the mechanism(s) responsible for DS-AD are yet to be defined, a highly salient finding is that an increased amyloid precursor protein (APP) gene dose is necessary (Lai and Williams, 1989; Zigman and Lott, 2007). Rarely is a person with DS partially trisomic for (human chromosome 21 HSA21) and carries only two copies of APP; these people demonstrate neither AD neuropathology nor dementia (Prasher et al., 1998; Doran et al., 2017). Studies in mouse models of DS show age-related degenerative features that also demonstrate dependence on increased APP gene dose (Salehi et al., 2006, 2009; Sawa et al., 2021). Accordingly, mouse models of DS can be used to define pathogenetic mechanisms and test treatments to prevent pathogenesis. Studies that link brain findings to biomarkers may prove especially useful.

Ts65Dn mice are widely used and serve as the best-characterized animal model of DS. The Ts65Dn mice are segmentally trisomic for a portion of mouse chromosome 16 which harbors a region syntenic to HSA21 that contains the murine gene for APP (Davisson et al., 1990). Ts65Dn mice have increased levels of full-length murine APP and its products, including Aβ40 and Aβ42 (Hunter et al., 2004). Ts65Dn mice demonstrate neuropathological changes and age-related behavioral changes that model DS-AD (see Belichenko et al., 2015, Table 1 for review). Additionally, the Ts65Dn mice have enhanced Tau expression likely due to the additional copy of Dyrk1A which increases Tau mRNA stability (Qian et al., 2013) and mediates the phosphorylation of Tau (Liu et al., 2008). Given age-related neuropathological findings and clinical biomarkers that define DS-AD, we undertook studies to characterize age-related changes in proteins linked to axonal structure, biomarkers of inflammation, and behavior. We found age-related changes in both Ts65Dn and 2N mice. Relative to 2N mice, Ts65Dn mice showed a consistent increase in Aβ40, insoluble pTau, and NfL that were correlated with deficits in learning and memory.

## 2. Materials and methods

### 2.1. Mice

Segmental trisomy 16 (Ts65Dn) mice (Davisson et al., 1993) were maintained on the B6/C3H background and diploid (2N) littermate mice served as controls. Mice were housed and genotyped as described before (Kleschevnikov et al., 2012). This PCR amplifies the breakpoint/addition region at the junction of chromosomes 16 and 17 in Ts65Dn mice. The primers listed were previously described by Duchon et al. (2011).

FW_Chr17Ts65Dn: 5′-GAC TTA GTA AGA GCA AGT GGC-3′ [Chr17 fwd.].

Rev_Chr16Ts65Dn: 5′-AGG TAG AAA GAT GTG AGG ACA C-3′ [Chr16 rev.].

wt_Chr17rev: 5′-GGG CAA CAC TGG ATC AAT C-3′ [wt-Chr17 rev.].

#### 2.1.1. Standard PCR for RD

The recessive retinal degeneration mutation 1 (RD1) is found in the Ts65Dn and background (B6EiC3SnF1/J) strains of mice. The RD/WT PCR checks for allelic variation at the Pdeb locus. The primers listed for RD1 are as follows:

RD2093: 5′-AAG CTA GCT GCA GTA ACG CCA TTT-3′ [RD primer].
RD2094: 5′-ACC TGC ATG TGA ACC CAG TAT TCT ATC-3′ [WT primer].
RD2095: 5′-CTA CAG CCC CTC TCC AAG GTT TAT AG-3′ [Common primer].

In addition, all mice were prescreened for Pde6brd1 homozygosity, a recessive retinal degeneration mutation that results in blindness (Bowes et al., 1993), and only animals free of retinal degeneration were used. Male mice, divided between two genotypes (2N controls & Ts65Dn), and age groups (7, 10, 13, and 16 months of age at the time of death) were used in this study (*N* = 13–18 per group for behavioral studies and *N* = 3–7 for biochemical analysis). Mice were housed 1–5 per cage in standard polycarbonate cages (19 × 31 × 13 cm) with Aspen Sani-Chip Bedding (#7090A Harlan Teklad) and a cotton fiber nestlet (Ancare), in a temperature-controlled room (21 ± 2°C), on a reversed 12 h light:12 h dark cycle (lights off at 8:00 AM). Mice had *ad libitum* access to standard chow (Teklad Global 18% Protein Rodent Diet, Envigo) and water, and cages were changed once weekly. All procedures were approved by The Scripps Research Institution’s Animal Care and Use Committee and met the guidelines of the NIH detailed in the Guide for the Care and Use of Laboratory Animals.

Experiments were conducted following the National Institutes of Health guidelines under protocols approved by the University of California San Diego (UCSD) Institutional Animal Care and Use Committees. The Ts65Dn and 2N mice reported herein served as vehicle-treated (PBS) controls as a preparatory study for future evaluation of vaccine immunization protocol/schedule. All mice were injected subcutaneously with PBS (200 μL/mouse) beginning on experiment day 0 for mice at ages 3-, 6-, 9-, or 12 months of age, and then on days 14, 28, 56, and 84 until the time of sacrifice (112 days from first injection) at 7-, 10-, 13-, or 16- months of age (Supplementary Figure S1). Separate groups of mice were used for behavioral testing from those used in the subsequent biochemical and histological analyses.

#### 2.1.2. Brain lysate

The left hemispheres were collected at sacrifice at UCSD and snap frozen at −80°C. To prepare total brain homogenates (total brain homogenate), the brain hemispheres were weighted and homogenized in 6 volumes/weight (mL/g) of ice cold-homogenization buffer [25 mM Tris/HCl pH 7.6, 150 mM NaCl, 1 mM EDTA, 1 mM EGTA containing phosphatase inhibitors, 30 mM NaF, 0.2 mM Na3VO4, 1 nM okadaic acid, 1 mM phenylmethylsulfonyl fluoride (PMSF), 5 mM Na4P2O7 and protease inhibitor cocktail (Complete TM, Roche)] and homogenized using a tissue homogenizer (Precellys 24). Samples were then aliquoted and stored at −80°C.

#### 2.1.3. Insoluble and soluble fractions

This method has been previously described (Theunis et al., 2013). Briefly, total brain homogenate was centrifuged for 30 min at 150,000 g at 4°C in 2 mL Beckman tubes. The supernatant (S1) was separated from the pellet and stored at −80°C. The pellet was further re-suspended in 500 μL of sucrose buffer (10 mM Tris–HCl (pH 7.4), 0.8 M NaCl, 10% sucrose, 1 mM EGTA, and 1 mM PMSF) and centrifuged for 10 min at 14,000 g at 4°C. The soluble fraction was then incubated for 1 h at room temperature in 1% sarkosyl and then centrifuged for 30 min at 150,000 g at 4°C. The supernatant (sarkosyl soluble fraction) was collected; the pellet (sarkosyl insoluble fraction) was then re-suspended in 50 μL of TBS buffer (10 mM Tris HCl (pH 7.4), 0.8 M NaCl) and both fractions were stored at −80°C until analysis.

#### 2.1.4. Quantification of pTau S396 and total Tau in total brain homogenate and total brain homogenate insoluble fraction by western blot

Proteins were separated using the JESS system (JESS Automated Western Blots with Simple Western; ProteinSimple, San Jose, CA, United States) with a 12–230 kDa Separation Module. First, 0.2–1 μg was mixed with master mix (Protein Simple) to achieve a final concentration of 1× sample buffer in the presence of fluorescent molecular weight markers and 40 mM dithiothreitol (DTT), the sample was denatured at 70°C for 10 min. Target proteins were immune-probed with primary antibodies (pTau S396 Abcam 109390, dilution 1:100) and total-Tau, BD biosciences 556319 (dilution 1:30) followed by HRP-conjugated secondary antibody (ProteinSimple, ready to use). Primary antibodies were diluted using an antibody diluent (ProteinSimple). The digital image was analyzed with Compass software (Protein Simple), and the quantified data of the detected proteins are reported as area (insoluble pTau) or corrected area (in TH samples) when the protein normalization reagent TAMRA was used.

#### 2.1.5. Quantification of total Tau, pTau396, pro-inflammatory markers, and Aβ by Meso Scale Discovery

The Meso Scale Discovery method was used to measure Tau, pTau, pro-inflammatory markers, and other relevant markers. The total Tau protein level in total brain homogenate samples was quantified using Kit # K151DSD-2 provided by Meso Scale Discovery following the manufacturer’s instructions. Samples (1/100 dilution) and calibrator were prepared in Meso Scale Discovery Blocker A 10% in 1x Meso Scale Discovery Tris Wash Buffer and 25 μL/well were added to the plate and incubated for 2 h with shaking at room temperature. Electrochemiluminescence (ECL) signals were measured with the Meso Scale Discovery SECTOR S 600 Instrument. The results, expressed in ng/mL, were obtained by back-calculation from the standard curve, with an unweighted four-parameter logistic regression model using the Meso Scale Discovery Discovery Workbench software 4.0. The total Tau level obtained by Meso Scale Discovery was normalized on the total brain homogenate protein concentration and reported in the graph as ng/mL.

Quantification of pTau396 in total brain homogenate or insoluble fraction of total brain homogenate was performed using Meso Scale Discovery. A Meso Scale Discovery Multi-Array 96 well small standard plate was coated with 10 μg/mL anti-Tau antibody (BD biosciences 556319) in Tris buffer (50 mM Tris pH7.6, 150 mM NaCl) and incubated overnight at 4°C. After washing the plates three times with 150 μL of Tris Wash Buffer (50 mM Tris pH7.6, 150 mM NaCl, 0.02% Tween-20), the blocking step with 1% Blocker A (Meso Scale Discovery, R93BA-4) in Tris Wash Buffer was performed for 1 h with shaking at room temperature. Total brain homogenate samples were diluted (dilution 1/100) in blocking solution (1% Blocker A in Tris Wash Buffer) and 25 μL/well added. Recombinant pTau protein (Signal Chem, T08-54 N) was used as a standard in 10 twofold dilutions starting at 1,000 ng/mL in blocking solution (1% Blocker A in Tris Wash Buffer). After incubating for 2 h with shaking at room temperature, the plates were washed three times with 150 μL of Tris Wash Buffer and 25 μL/well of anti-pTauS396 (Abcam 109390) antibody conjugated to SULFO-TAG in blocking solution (dilution 1/100), was added and incubated for 1 h with shaking at room temperature. Electrochemiluminescence (ECL) signals were measured right after the addition of the substrate, Read buffer (Meso Scale Discovery R92TC-2, diluted 2x in MilliQ water) with the Meso Scale Discovery SECTOR S 600 Instrument. The results, expressed in pg/mL, were obtained by back-calculation from the standard curve with an unweighted four-parameter logistic regression model using the Meso Scale Discovery Discovery Workbench software 4.0. In total brain homogenate samples, the pTau S396 level obtained by Meso Scale Discovery was normalized on the total brain homogenate protein concentration and reported in the graph as ng/mL.

Quantification of pro-inflammatory markers in total brain homogenate by Meso Scale Discovery. The inflammatory cytokines were measured in total brain homogenate using V-PLEX Plus Pro-inflammatory Panel 1Mouse Meso Scale Discovery Kit #K15048G following the manufacturer’s instructions. Total brain homogenate was centrifuged for 2 min at 2,000 g (4°C) and undiluted supernatant was applied to the kit. ECL signals were measured with the Meso Scale Discovery SECTOR S 600 Instrument. The concentration of each cytokine was calculated using a standard included in the kit with the Meso Scale Discovery Discovery Workbench software 4.0, normalized on the total protein concentration, and reported as fg in the graph.

Quantification of Aβ in plasma and total brain homogenate by Meso Scale Discovery. Approximately 100–200 μL of blood was collected from the tail using microvette CB 300 LH tubes from Starstedt and stored in 1 mL eppendorf tubes. Eppendorf tubes were centrifuged at 14,000 g in an Eppendorf centrifuge for 5 min. Plasma was stored at −80°C until analyzed. For the quantification of Aβ 1–40 levels in plasma, Meso Scale Discovery Aβ Peptide Panel 1 Kit # K150SJG was used following the manufacturer’s instructions. Samples, 25 μL/well (dilution 1/2), and a calibrator diluted in Diluent 35 were added to the detection antibody and incubated with shaking at room temperature for 2 h. Meso Scale Discovery Read Buffer was added after washing the plates and the plates were read at once by the Meso Scale Discovery SECTOR S 600 Instrument. The results, expressed in pg/mL, were obtained by back-calculation from the standard curve with an unweighted four-parameter logistic regression model using the Meso Scale Discovery Discovery Workbench software 4.0. In total brain homogenate samples, the Aβ1-40 level obtained by Meso Scale Discovery was normalized on the total brain homogenate protein concentration and reported in the graph as ng/mL.

#### 2.1.6. Quantification of neurofilament light chain (NfL) in plasma by Simoa

The NfL protein concentration in plasma was measured using Quanterix’s Simoa Human Neurofilament Light Chain Kit HD-1/HD-X, which was cross-reactive with murine NfL epitopes, following the manufacturer’s instructions at PsychoGenics (US). Samples were diluted 1:15 in assay diluent and evaluated in duplicate. Sample values (AEB units) were measured and NfL concentrations (pg/mL) were interpolated from a calibration curve. Reported NfL concentrations were dilution corrected. Plasma was collected from mice at 5.5, 8.5, 11.5, and 14.5 months of age, as described above (Supplementary Figure S1).

### 2.2. Histology for total Aβ in the frontal cortex and hippocampus

The right hemisphere was collected and fixed either by immersion or perfusion. Then the brains were post-fixed in 4% PFA for 24 h and stored in 30% sucrose for at least 24 h. Tissue was stored at +4°C in cryoprotectant buffer (Sucrose 150 g, 0.1 M PB 200 mL, Ethylene glycol 150 mL, add more 0.1 M PB to reach a final volume of 500 mL) and shipped to PsychoGenics (Czechia). A systematic random set of 5 sagittal sections from 5 different mediolateral levels per mouse was labeled for Aβ, using an antibody to residues 1–4 of Aβ (MOAB2, Abcam 126649, dilution 1/1,000). Sections were counterstained with DAPI to visualize nuclei. Antibody binding was visualized using a highly cross-absorbed fluorescently labeled secondary antibody (Abcam 150106, dilution 1/500). All antibodies were diluted in antibody diluent (Dako, S 302283–2), and unspecific endogenous IgG binding was blocked with M.O.M. (Vector MKB 2213-1) before primary incubation. Entire brain sections were imaged on a Zeiss AxioScan.Z1 slide scanner microscope. Quantifications using the stored ROIs were done using Image Pro Premier software (v 9.1 or higher).

### 2.3. Behavioral tests

Mice were transferred from UCSD to TSRI, acclimated for 7–10 days, then tested in the order described below, which was devised to minimize carry-over effects, and with at least 4 days between tests. In addition, all testing occurred between 1 and 5 h following the lights off, i.e., in the early portion of the animals’ active phase.

#### 2.3.1. Y maze test

Spontaneous alternation behavior, a measure of spatial working memory, exploratory behavior, and responsiveness to novelty, was tested using a Y maze with 34 × 8 × 14-cm arms. Each mouse was evaluated in a single 5-min trial and spontaneous alternations, sets of three unique arm choices, were recorded. Because mice have the opportunity to do repeated entries into a single arm, there is a chance performance level of 22% (2/9) for spontaneous alternations. Healthy young C57BL6/J mice typically make 50–70% spontaneous alternations in this test.

#### 2.3.2. Marble burying test

Mice were placed individually in a standard mouse cage having bedding that was 5 cm in depth, with 20 small marbles arranged in 4 evenly spaced rows of 5 on top of the bedding material. After 30 min mice were removed and the number of marbles buried (at least 2/3 covered by bedding) was determined. Increased marble burying is associated with increased anxiety-like behavior in tests of anxiety medications (Albelda and Joel, 2012) and this behavior is hypothesized to reflect a response to novelty and affective state (Deacon, 2006) be a measure of repetitive behavior, perseverative and/or stereotyped-like behavior, obsessive–compulsive-like behavior (Londei et al., 1998), and is sensitive to hippocampal damage (Deacon and Rawlins, 2005).

#### 2.3.3. Open field test

This test measures how animals respond when introduced into a brightly illuminated open arena (Crawley, 1999). It is a classical test used to measure anxiety-like responses of rodents exposed to stressful environmental stimuli (brightly illuminated open spaces) as well as to capture spontaneous activity measures. The apparatus is a square white Plexiglas (50 × 50 cm) open field illuminated to 450 lux in the center. Each animal was placed in the center of the field and several behavioral parameters (distance traveled, velocity, center time, frequency in the center) were recorded during a 10-min observation period and analyzed using Noldus Ethovision XT software.

#### 2.3.4. Novel object recognition test

Animals decrease the exploration of their environment as it becomes familiar. However, they will show renewed interest if there is a change, which involves hippocampal circuitry (Berlyne, 1950; Ennaceur and Delacour, 1988; Winters et al., 2004; Mumby et al., 2005; Lieben et al., 2006). In order to evaluate short-term memory, mice were individually habituated to an open field for 5 min and were evaluated with two identical objects placed in the field using 2 trials of 5 min each with one-minute elapsing between time trials. After this, and following another one-minute delay, the mice were evaluated for novel object recognition by assessing object contact time when a novel object replaced one of the familiar objects.

#### 2.3.5. Conditioned fear test

In this procedure, mice learn to associate a novel environment (context) and a previously neutral stimulus (conditioned stimulus, tone) with an aversive foot shock stimulus (Maren, 2001). It allows for the assessment of both hippocampus-dependent and amygdala-dependent learning processes in the same mouse (Rudy et al., 2004; Kenney and Gould, 2008). Briefly, mice were habituated to the system (Freeze Monitor, Med Associates, VT) to measure baseline freezing behavior on day 1 and then on day 2 were conditioned with two 0.6 mA foot shocks given in the final 2 s of cue (light and tone) exposure. On day 3 contextual conditioning was examined by measuring freezing behavior associated with the box and then on day 4 the box was camouflaged, and the freezing response was measured before and during cue exposure.

### 2.4. Statistical analysis

Data were assessed for normality and homogeneity of variances before two-way or three-way parametric ANOVA statistical testing for differences in genotype (Ts65Dn vs. 2N) and age, or behavioral parameter and age followed by Tukey’s test for *post hoc* analysis except for levels of pTau S396. in the insoluble fraction where a T-test was used to evaluate the differences between two groups. Statistical significance is reported throughout the manuscript text with positive *post hoc* findings also reported on the figure. Statistically significant ANOVA values are also reported in the figure legends. All statistics were performed using GraphPad Prism 9.0 (GraphPad Software, La Jolla, CA).

## 3. Results

### 3.1. Age-related changes in Aβ levels in plasma and brain in Ts65Dn mice

**Figure 1:**
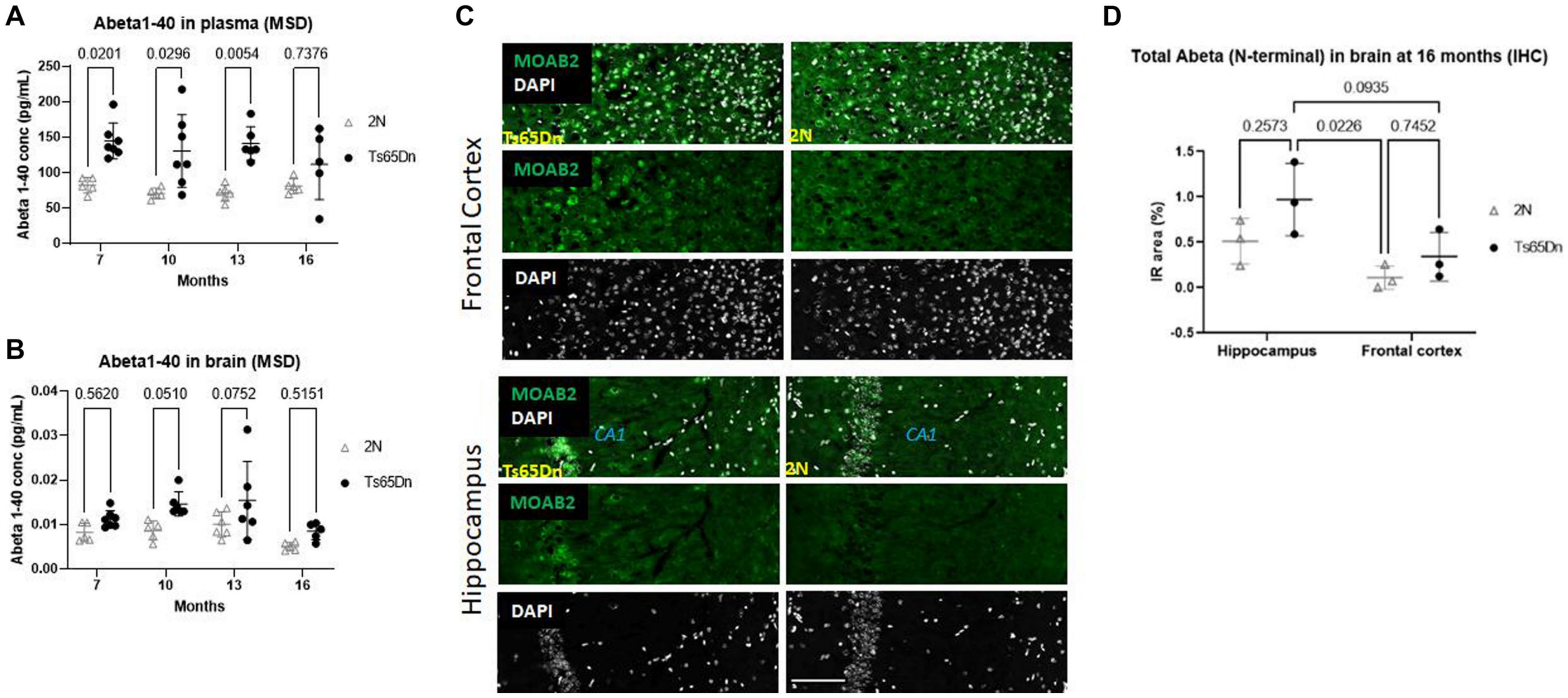
Aβ 1–40 and Aβ protein level in plasma and brain at different ages by Meso Scale Discovery and IHC. **(A)** Aβ 1–40 protein level in plasma and **(B)** in brain total homogenates normalized on the total protein concentration at different ages of 2N and Ts65Dn mice. Results are expressed as individual values and mean ± SD. ANOVA revealed a significant difference between Aβ 1–40 plasma levels in Ts65Dn and 2N mice (*p* < 0.0001) independent of age (*p* = 0.5769; top panel). ANOVA of Aβ40 levels in the brain total homogenate was also significantly increased in Ts65Dn compared to 2N mice (*p* = 0.0004), age was also a significant source of variation (*p* = 0.005). **(C)** Images and **(D)** quantification of total Aβ immunoreactivity in the hippocampus and frontal cortex in 2N and Ts65Dn mice at 16 months of age. Results are expressed as individual values and mean ± SD Immunoreactive (IR) area % (as the percentage of the area with a positive signal for Aβ staining with an antibody binding the N-terminal part of Aβ). ANOVA evaluation of total Aβ in the hippocampus compared to the frontal cortex was statistically significant (*p* = 0.0128); genotype was not a statistically significant factor (*p* = 0.0641). Scale bar = 100 μm.

Prior studies in the Ts65Dn mouse demonstrated changes in the levels of *APP* mRNA and its products, including Aβ peptides (Chen et al., 2021). We examined mice at ages ranging from 7- to 16 months to examine age-related changes in plasma and brain levels of Aβ40, the most abundant Aβ species. On the contrary, the concentration of Aβ1-42 and Aβ1-38 peptide levels were not measurable (below the limit of quantification of the Meso Scale Discovery assay). In plasma, Aβ1-40 levels were significantly increased in Ts65Dn mice compared to 2N mice (*p* < 0.0001) independent of age (*p* = 0.5769; Figure 1A). The increases at ages 7 to 13 months modestly exceeded *APP* gene dose (Ts65Dn/2N Aβ40 levels; at 7 months ~1.7; at 10 months ~1.8; at 13 months ~1.9); at 16 months the ratio decreased to ~1.5. Following a *post hoc* analysis, there were significant increases in Aβ1-40 levels in Ts65Dn mice compared to wt mice at 7 (*p* = 0.0201), 10 (*p* = 0.0296), and 13 (*p* = 0.0054) months of age (Figure 1A); at 16 months the increase was not statistically significant. Aβ40 levels present in the total homogenates of the brain were also significantly increased in Ts65Dn mice (*p* = 0.0004; Figure 1B). Interestingly, age was also a significant source of variation (*p* = 0.005; Figure 1B). Post-hoc analysis did not identify a significant difference at any specific age, although 10 mos were nearly statistically significant (*p* = 0.0510). Nevertheless, like plasma measures, Aβ1-40 levels were higher in Ts65Dn versus 2N mice at all ages and the levels at younger ages exceeded those at 16 months. Immunohistochemical analysis of total Aβ in the frontal cortex and hippocampus in 2N and Ts65Dn mice were quantitatively assessed for the immunoreactive area (Figures 1C,D). Due to the limited number of samples (*N* = 3) only a qualitative inspection was performed; it suggested a trend of a higher level of Aβ in the frontal cortex compared to the hippocampus. It can be concluded that increased APP gene dose results in an increase in Aβ in both the brain and plasma of Ts65Dn mice.

### 3.2. Age-related changes in Tau levels in the brain in the Ts65Dn mice

**Figure 2:**
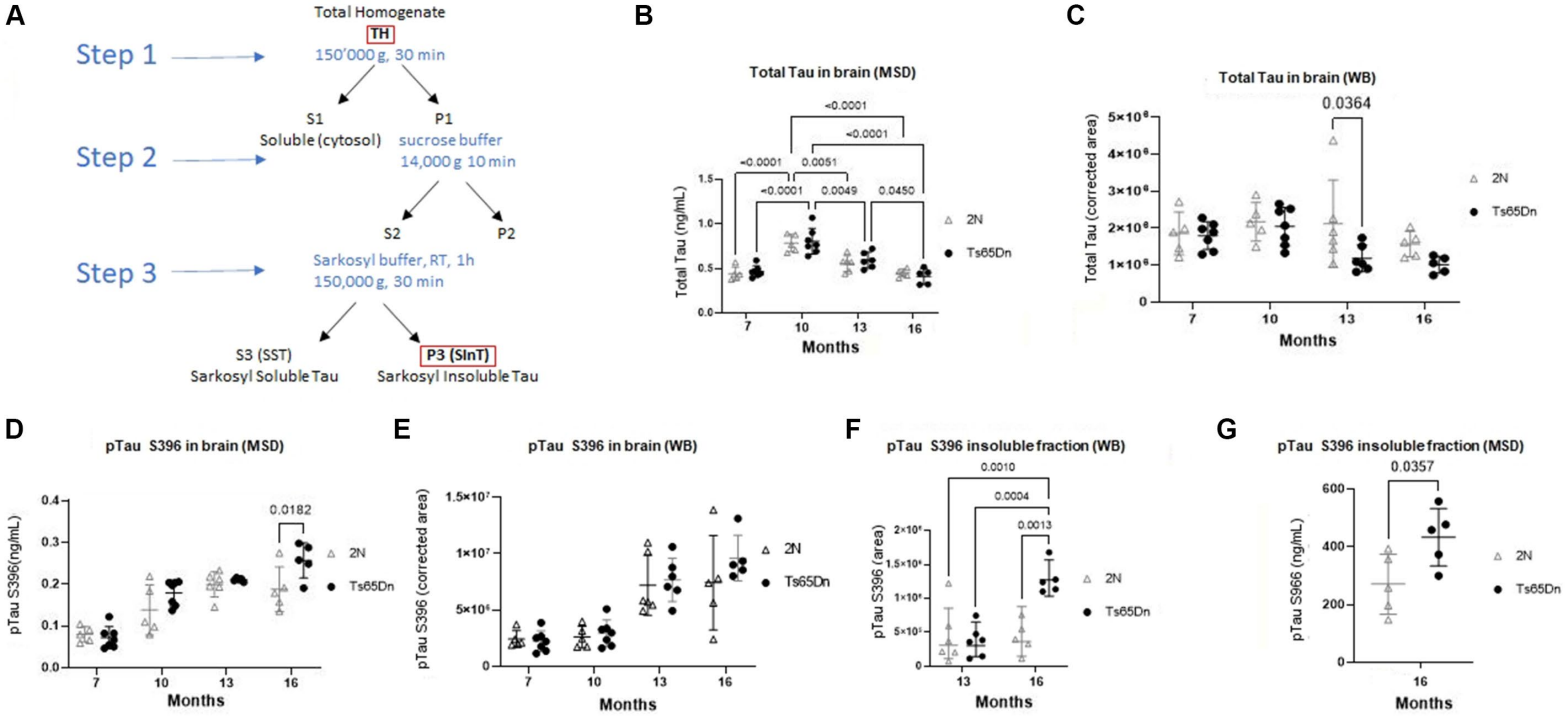
Total Tau and pTau S396 protein level in Ts65Dn and 2N mouse brain total homogenate and insoluble fraction at different ages by Meso Scale Discovery and WB. **(A)** Schematic representation of the fractions used for evaluation. The total homogenate was used for measuring total Tau and pTau levels. P3-SinT was used for the Sarkosyl insoluble Tau fraction. **(B)** Total Tau measured by Meso Scale Discovery. Age had a significant effect on levels of total Tau (*p* < 0.0001). **(C)** Total Tau as measured by WB. Age had a significant effect (*p* = 0.0218) as did genotype (*p* = 0.0218). **(D)** pTau S396 protein level in total brain homogenates of 2N and TS65Dn mice at different ages as measured by Meso Scale Discovery. Age (*p* < 0.0001) and genotype (*p* = 0.0228) were significant factors for pTau S396 levels. **(E)** WB evaluation of pTau S396 protein level in total brain homogenates of 2N and TS65Dn mice at different ages measured. Age (*p* < 0.0001) was a significant factor for pTau S396 levels. The concentrations of pTauS396 obtained by Meso Scale Discovery were normalized on the total protein concentration, while the results obtained by WB were normalized on the total protein concentration and expressed as the corrected area. **(F)** pTau S396 protein level in Sarkosyl-insoluble fraction of the 13- and 16-month-old cohort measured by WB. ANOVA indicated a significant effect of genotype (*p* = 0.0066), age (*p* = 0.0024), and interaction of age and genotype (*p* = 0.0066). **(G)** pTau S396 protein level in Sarkosyl-insoluble fraction of the 16-month-old cohort measured by Meso Scale Discovery. Results are expressed as individual values and mean ± SD.

Tau pathology is characteristic of AD and DS-AD, and in AD these changes are linked to changes in cognition (Braak et al., 2006). Accordingly, we undertook studies of total Tau and pTauS396 in the brains of Ts65Dn and 2N mice. Total Tau levels were examined using Meso Scale Discovery and western blot analysis at 7-, 10-, 13-, and 16 months of age (Figures 2B,C). As measured using Meso Scale Discovery, two-way ANOVA analysis identified a significant effect of age on levels of total Tau (Figure 2B; *p* < 0.0001). Interestingly, both 2N and Ts65Dn levels of total Tau peaked at 10 months of age as measured by both Meso Scale Discovery (Figure 2B) and WB (Figure 2C and Supplementary Figure S2). Total Tau levels at 10 months exceeded those at 7 months (*p* < 0.0001 for both 2N and Ts65Dn mice, respectively), 13 months (*p* = 0.0051 and 0.0049 for 2N and Ts65Dn mice, respectively), and at 16 months of age (*p*s < 0.0001 for both 2N and Ts65DN mice; Figure 2B). There was no significant effect of genotype on total Tau levels (*p* = 0.6477). Total Tau levels, as measured using WB, revealed a significant effect of age (*p* = 0.0208) and genotype (*p* = 0.0218), although there was no effect of the interaction of age and genotype (*p* = 0.2564; Figure 2C). Sidak’s multiple comparison *post hoc* analysis identified a significant difference at 13 months of age between 2N and Ts65Dn mice (*p* = 0.0364; Figure 2C).

The Tau protein is prone to misfolding and aggregation caused by phosphorylation causing conformational changes, which has been associated with a breakdown in the microtubules in late-stage AD. pTau S396 can lead to a change in the conformation of Tau, which makes the protein more prone to aggregation. While Liu et al. have previously shown higher levels of pTau in Ts65Dn mice at 15 months of age compared to 2N mice age, to our knowledge, nobody showed the level of pTau over time. Therefore, we evaluated pTau S396 and found in both 2N and Ts65Dn mice significant age-related increases in total brain homogenates (TH) (Figure 2A) pTau S396 levels as measured by Meso Scale Discovery (Figure 2D) and WB (Figure 2E and Supplementary Figure S3). Age was a significant factor for pTau S396 levels measured in both Meso Scale Discovery and WB (*p* < 0.0001, for both) and genotype was a significant factor for pTau S396 levels measured by Meso Scale Discovery (*p* = 0.0228). *Post hoc* Tukey’s multiple comparisons tests identified significant increases in pTau S396 levels measured by Meso Scale Discovery in Ts65Dn mice at 10 (*p* < 0.0001), 13 (*p* < 0.0001), and 16 (*p* < 0.0001) months of age compared to 7 months of age, and at 16 months of age compared to 10 months of age (*p* = 0.0053). In 2N mice, there were significant increases in the levels of pTau S396 at 13- (*p* < 0.0001) and 16- (*p* = 0.0002) months of age compared to 7 months of age. Similarly, *post hoc* Tukey’s multiple comparisons test of pTau S396 levels measured by WB identified significant increases in pTau 396 levels measured by Meso Scale Discovery in Ts65Dn mice at 13 (*p* < 0.0028) and 16 (*p* < 0.0001) months of age compared to 7 months of age, and at 13 (*p* = 0.0043) and 16 (*p* < 0.0001) months of age compared to 10 months of age. In 2N mice, there were significant increases in the levels of pTau396 at 13- (*p* = 0.0002) and 16- (*p* = 0.0006) months of age compared to 7 months of age, as well as at 13- (*p* = 0.0010) and 16- (*p* = 0.0036) months of age compared to 10 months of age.

In contrast to increases in soluble pTau S396 in both Ts65Dn and 2N mice, the changes in insoluble (Figure 2A) pTau S396, which are characteristic of the disease state, were only seen in Ts65Dn mice. Statistical analysis indicated a significant effect of genotype (*p* = 0.0066), age (*p* = 0.0024), and interaction of age and genotype (*p* = 0.0066). Indeed, while the two genotypes did not differ at age 13 months, at 16 months of age there was a significant increase in the levels of pTau S396 in the insoluble fraction from Ts65Dn mice compared to 2N mice as measured by WB (*p* = 0.0013; Figure 2F and Supplementary Figure S4). Moreover, there was a significant increase in the levels of pTau S396 in the insoluble fraction from Ts65Dn mice at 16 months of age compared to 13 months of age (*p* = 0.0004). Using Meso Scale Discovery, we confirmed the increase in Ts65Dn versus 2N at 16 months of age (*p* = 0.0357; Figure 2G). Overall, pTau increased over time for both Ts65Dn and 2N until 13 months of age. From 13 to 16 months, the increase was detected only in the total homogenates of Ts65Dn mice, and this increase is even more clear in the insoluble fraction.

### 3.3. Age-related changes in inflammation

**Figure 3:**
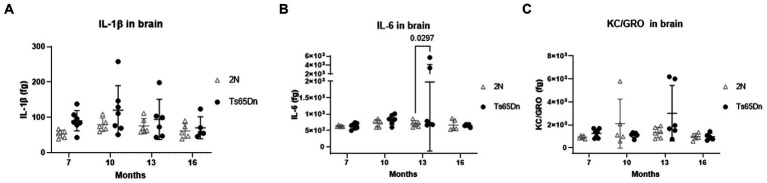
IL-1β, IL-6, and KC/GRO protein levels in brain total homogenate at different ages by Meso Scale Discovery. IL-1β **(A)**, IL-6 **(B)**, and KC/GRO **(C)** levels (femtogram (fg) of cytokine normalized on the total protein concentration) in total brain homogenates of 2N and Ts65Dn mice at different ages by Meso Scale Discovery. IL-1β was significantly affected by genotype (*p* = 0.0305). Results are expressed as individual values and mean ± SD.

In DS, several gene products may contribute to increased immunoreactivity both systemically (Zhang et al., 2017) and in the brain (Wilcock, 2012; Flores-Aguilar et al., 2020). Indeed, recent findings point to a role for the extra copy of several interferon receptor genes on HSA21 as predisposing people with DS to autoimmune disorders. Moreover, the role of inflammatory events contributing to AD pathogenesis in neurotypical individuals has further increased interest in immune-mediated events in the context of AD in DS. To ask if there are effects of age and genotype on markers of inflammation, we measured levels of IL-1β, IL-6, and KC/GRO which is involved in neutrophil activation. Levels of IL-1β, IL-6, and KC/GRO were measured in the brains of Ts65Dn and 2N mice at 7-, 10-, 13-, and 16 months of age (Figure 3). While there was a high intra-group variability, IL-1β was significantly affected by genotype (*p* = 0.0305). Although *post hoc* analysis did not reveal any significant differences by genotype, IL-1β averages in Ts65Dn mice exceeded those for 2N mice at ages 7 through 13 months. In contrast, neither IL-6 (*p* = 0.1144 and 0.1513) nor KC/GRO levels (*p* = 0.0709 and 0.4703) were significantly affected by age or genotype, respectively.

### 3.4. Neurofilament L increases with age and genotype

**Figure 4:**
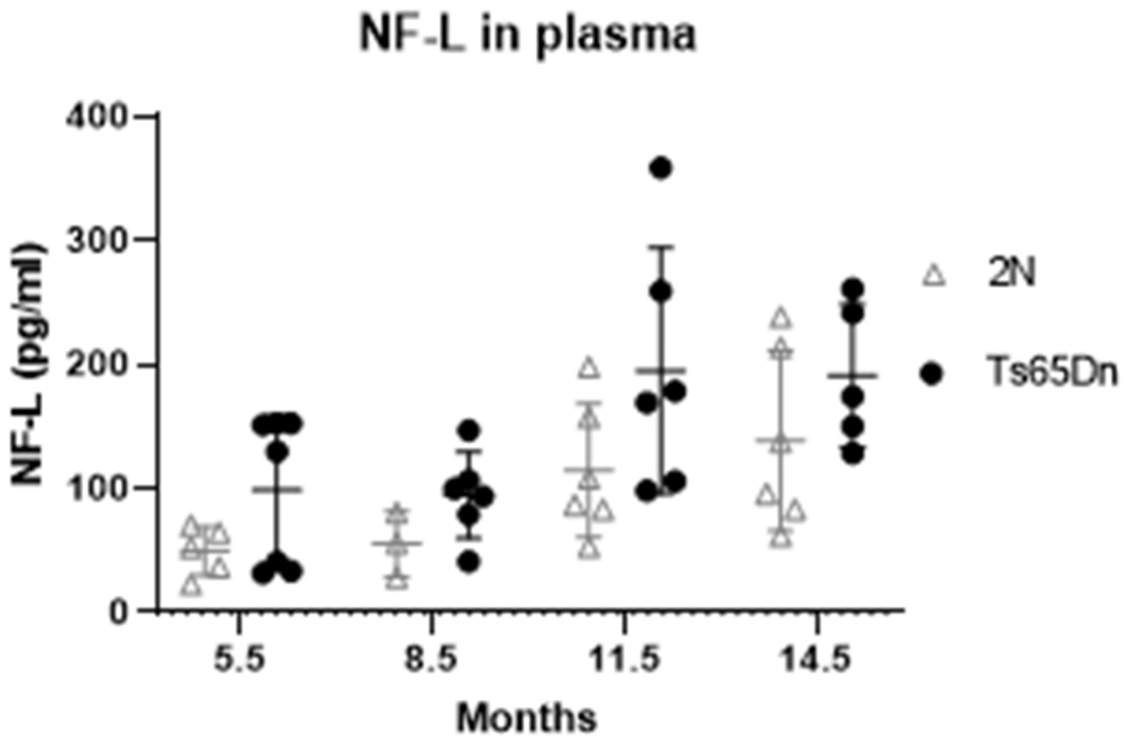
Neurofilament light chain (NfL) protein level in plasma at different ages by Simoa. NfL level in plasma of 2N and Ts65Dn mice at different ages by Simoa. ANOVA analysis revealed a significant effect of age (*p* = 0.0008) and genotype (*p* = 0.0058) on NfL protein levels. Results are expressed as individual values and mean ± SD.

Among the most promising biomarkers for disease progression is NfL. This type IV intermediate filament protein is expressed exclusively in neurons together with two other neurofilament proteins, medium and heavy (NfM and NfH, respectively), and interacts with microtubules to stabilize the axonal cytoskeleton (reviewed in Julien, 1999). While NfL is normally released from axons, in people with AD NfL is released in much larger amounts (Mattsson et al., 2017). Recent studies documented increases in plasma in those with AD as well as with increasing age in those with DS. The increases are viewed as resulting from a breakdown of axons and possibly synapses, thereby reflecting increased degeneration of neurons. NfL levels in plasma (Figure 4) demonstrated significant effects of age (*p* = 0.0008) and genotype (*p* = 0.0058). NfL levels increased in both Ts65Dn and 2N mice with increasing age, with averages higher in Ts65Dn mice at all ages; however, there was no interaction between age and genotype (*p* = 0.8808). Nevertheless, *post hoc* Tukey’s multiple comparisons identified a significant increase in NfL levels in Ts65Dn mice at 11.5 months of age compared to both 8.5 (*p* = 0.0344) and 5.5 (*p* = 0.0361) months of age.

### 3.5. Effects of age and genotype on behavior

**Figure 5:**
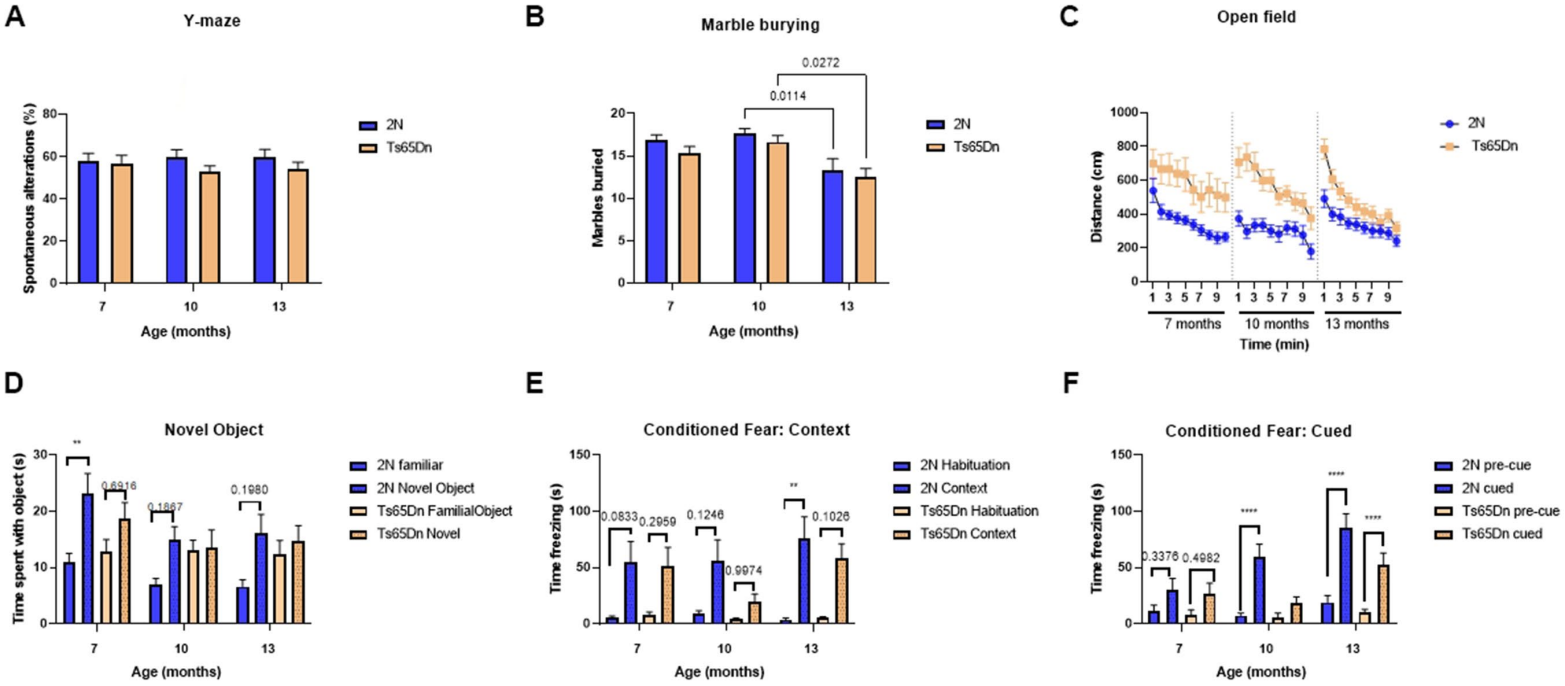
Behavioral evaluation and memory function of 2N and Ts65Dn mice at different ages. Behavioral evaluation of age-and genotype-related changes was assessed. **(A)** Y-maze percent spontaneous alterations at 7-, 10-, and 13 months of age. **(B)** Number of marbles buried in the Marble burying test at 7-, 10-, and 13 months of age. ANOVA analysis revealed a significant effect of age (*p* = 0.0149) on the number of marbles buried. **(C)** Distance traveled over 10 min in the open field test at 7-, 10-, and 13 months of age. There were significant effects of age (*p* < 0.0001), genotype (*p* < 0.0001), time (*p* < 0.0001), and the interaction of age and genotype (*p* < 0.0001) on the distance traveled in the open field test. **(D)** Contact time with a familiar or novel object in the novel object recognition test at 7-, 10-, and 13 months of age. The type of object (familiar vs. novel) consistently had a significant effect on contact time (*p* < 0.0001), age was a significant factor (*p* = 0.0490), and there was a significant interaction of genotype and type of object (*p* = 0.0128). **(E)** Time spent freezing in the conditioned fear context test at 7-, 10-, and 13 months of age. There was a significant effect of context on the time spent freezing (*p* < 0.0001). **(F)** Time spent freezing in the conditioned fear cued test at 7-, 10, and 13 months of age. Results are expressed as mean ± SEM. ANOVA analysis revealed a significant effect of age (*p* = 0.0019), genotype (*p* = 0.0067), cue (*p* < 0.0001), the interaction of age and cue (*p* < 0.0001), genotype and cue (*p* = 0.0009), and the interaction of age, genotype, and cue (*p* = 0.0467). Statistical analysis was performed using 2- or 3-way ANOVA with multiple comparison *post hoc* tests. *p < 0.05; **p < 0.01; ****p < 0.0001. Specific *p*-values for relevant comparisons are included in the results section. At 7 months of age, the number of mice/group was as follows: 2N = 15; Ts65Dn = 14. At 10 months of age, the number of mice/group was as follows: 2N = 16; Ts65Dn = 15. At 13 months of age, the number of mice/group was as follows: 2N = 14; Ts65Dn = 13.

Ts65Dn and 2N mice were evaluated behaviorally using the Y-maze, marble burying, open field, novel object recognition task, and cued and contextual fear conditioning tasks (Figure 5). In the Y-maze (Figure 5A) there was no significant effect of age (*p* = 0.9337), genotype (*p* = 0.1137), or interaction of age and genotype (*p* = 0.6847). In the marble burying test (Figure 5B), there was a significant effect of age (*p* = 0.0149) with older mice burying fewer marbles. There was no effect of genotype (*p* = 0.1122) or interaction of age and genotype (*p* = 0.7062). Interestingly, in the open field test (Figure 5C) there were significant effects of age (*p* < 0.0001), genotype (*p* < 0.0001), time (*p*<0.0001), and the interaction of age and genotype (*p* < 0.0001). As expected, Ts65Dn mice consistently traveled farther than the 2N mice; over the 10-min observation period there was a significant decrease in distance traveled across all groups at 7, 10, and 13 months of age (Figure 5C). In the novel object recognition task (Figure 5D) the type of object (familiar vs. novel) consistently had a significant effect on contact time (*p* < 0.0001) at 7-, 10-, and 13-months of age. While genotype did not have a significant effect (*p* = 0.6774) on total contact time, there was a significant interaction of genotype and type of object (value of *p* = 0.0128) with 2N mice spending more time in contact with a novel object compared to the familiar object. Additionally, age was also a significant factor (*p* = 0.0490) with mice spending less time interacting with the objects with increasing age (Figure 5D). Ts65Dn mice failed to distinguish between the two types of objects at any age (Figure 5D); the deficit in contacting the novel versus the familiar object was most evident at 10 and 13 months of age.

Finally, in the contextual fear test, there was a significant effect on the time spent freezing (*p* < 0.0001) with mice freezing more when placed in the same context associated with the foot shock (Figure 5E). None of the other factors had a significant effect including age (*p* = 0.2910), genotype (*p* = 0.1743), the interaction of age and genotype (*p* = 0.4918), age and context (*p* = 0.1649), genotype and context (*p* = 0.1645), or age, genotype, and context (*p* = 0.7418). However, in the cued portion of the contextual fear test (Figure 5F), there was a significant effect of age (*p* = 0.0019), genotype (*p* = 0.0067), cue (*p* < 0.0001), the interaction of age and cue (*p* < 0.0001), genotype and cues (*p* = 0.0009), and the interaction of age, genotype, and cue (*p* = 0.0467). Interestingly, with age, the amount of time the 2N mice spent freezing significantly increased in response to the cues (*p* = 0.008 for cued 7- vs. 13- months and *p* < 0.0001 for cued 10- vs. 13- months); however, the Ts65Dn mice did not show the same effect (Figure 5F; *p* = 0.4596 for cued 7- vs. 13- months and *p* = 0.0668 for cued 10- vs. 13-months). In Ts65Dn mice there was no significant increase in time spent freezing, pre-cue vs. cued, at either 7 (*p* = 0.4982) or 10 months of age (*p* = 0.9248), although there was a significant difference at 13 months of age (*p* < 0.0001).

## 4. Discussion

The goal of the present study was to characterize the effect of age on genotype-induced changes in biochemical phenotypes and behavioral outcome measures in 2N and Ts65Dn mice. We observed age-related and genotype-linked differences in the levels of Aβ, total Tau, pTau, and NFL. Age-related behavioral changes were detected in the marble burying task, and age and genotype effects in the open field, novel object recognition, and conditioned fear-cued response. Finally, we showed that genotype-induced changes are reflected in the average increases in Ts65Dn versus 2N for Aβ at all ages, for pTau at the oldest age, for insoluble pTau at the age of 16 months, and NfL at all ages. These changes are relevant to AD in DS since people with DS show abnormally high levels of Aβ and pTau and changes in behavior and cognition in those diagnosed with dementia (Ballard et al., 2016). Except for Aβ and insoluble pTau, the other biochemical markers did not clearly distinguish Ts65Dn and 2N mice and even they showed modest differences. Nevertheless, the correlations with genotype raise the possibility that such age-related changes contribute to axonal injury (NfL) and deficits in synaptic function as revealed in novel object recognition and the conditioned fear-cued response.

### 4.1. Aβ and cytoskeleton markers

As expected, Ts65Dn mice had significantly elevated levels of Aβ1-40 in the plasma and the brain compared to 2N mice. Plasma levels of Aβ1-40 were increased in Ts65Dn vs. 2N mice independent of age, findings correlated with the extra copy of mouse *App*. In the brain, Aβ1-40 was also increased in Ts65Dn vs. 2N mice, but interestingly we found an age effect where levels of Aβ1-40 peaked at 10 months of age before declining at 16 months of age. In addition to the expected genotype-related increased protein levels, age-related increased levels of Aβ1-40 are in line with previously reported in Ts65Dn mice, with increases in APP levels from 2 to 6 months of age to 10–12 months of age (Choi et al., 2009; Ahmed et al., 2017). The mechanism by which Aβ1-40 decreases at older ages is unknown but could be due to changes in APP gene expression at the levels of transcription, translation, or processing, topics not addressed herein.

Next, we measured the levels of total Tau and pTau. While the *MAP Tau* gene is located on HSA17, there are only two copies of these genes. However, Tau expression and post-translation modification are regulated by *DYRK1A (*Liu et al., 2008; Qian et al., 2013), andTs65Dn mice contain an extra copy of this gene. Previous research has implicated the extra copy of *DYRK1A* in the hyperphosphorylation of Tau (Sheppard et al., 2012; Qian et al., 2013). An increase in total Tau, as measured by Meso Scale Discovery, was detected between 7 and 10 months with reductions at 13 and 16 months of age. Western blotting of the same samples also showed decreases in the average levels of total Tau after 10 months. Others have also reported increases in total Tau from 6- to 12 months of age in these mice (Ahmed et al., 2017). In contrast to the findings for total Tau, pTauS396 was increased after age 10 months by both Meso Scale Discovery and Western blotting. The most striking finding regarding Tau was the increase in pTauS396 in the insoluble fraction between ages 13 and 16 months; at 16 months, the levels in the Ts65Dn brain exceeded by ~2-fold those in the 2N brain. The age-related increase in insoluble pTau in Ts65Dn but not 2N mice gives evidence for increasing Tau pathology of the type present in DS-AD.

Neurofilament light (NfL) is a scaffolding cytoskeleton protein (Strydom et al., 2013) and blood concentrations of NfL correlate well with axonal damage in neurological disorders, including in AD (Mattsson et al., 2017; Weston et al., 2017) and DS (Strydom et al., 2018). People with DS have increased levels of plasma NfL (Strydom et al., 2018). In our study, Ts65Dn mice recapitulated this DS phenotype across both increasing age and genotype. This finding suggests that axonal injury is age-related in both genotypes but is more severe in Ts65Dn mice, especially in those less than 12 months old.

### 4.2. Inflammation

Inflammation is a hallmark of neurodegeneration. In our study, we showed a significant increase in IL-1β and a non-significant increase in IL-6 except at a single age. While a previous study showed a significant increase in IL-1β and IL-6 in Ts65Dn, our earlier investigation also failed to find a significant increase in IL-6 in Ts65Dn mice compared to 2N mice. The evidence in children with DS as to whether IL-6 levels are increased is conflicting, and the evidence in adults with DS while limited, suggests there is no difference compared to controls. KC/GRO (keratinocyte-derived chemokine/growth-regulated oncogene) is a protein biomarker of inflammation that is increased in AD (reviewed in Korbecki et al., 2022); however, it was not increased with age or genotype in our study.

### 4.3. Behavior

The marble burying test is a measure of repetitive and perseverative behavior with more marbles buried suggesting an increase in obsessive–compulsive-like behaviors. There was no difference in the number of marbles buried between Ts65Dn and 2N mice at 7, 10, or 13 months of age. This indicates that there is no change in the frequency of repetitive or stereotypic behavior in Ts65Dn mice, unlike TTS mice which have been previously reported to have a decrease in marble-burying behavior. Obsessive–compulsive behavior and anxiety are characteristic of children with DS (Evans and Gray, 2000). In adult individuals with DS, however, the prevalence rate for obsessive–compulsive disorder is not different compared to controls (0.8–4.5%) vs. the general population (1–4%) (Douglass et al., 1995). Indeed, using a very similar marble burying test, a standard approach for estimating obsessive–compulsive behavior (Londei et al., 1998), we have previously observed no evidence for increases in obsessive–compulsive behavior in Ts65Dn vs. WT mice (Belichenko et al., 2015). On the other hand, increased activity in the open field test has been well documented for Ts65Dn mice. We have similarly found increased activity at 7, 10, and 13 months of age. We have previously hypothesized that the hyperactivity could be due to biochemical abnormalities in the hippocampus, changes in cholinergic neurotransmission, or a loss of working memory which leads to an increased exploration of the area. The novel object recognition test involves the dentate gyrus and CA1/CA3 regions of the hippocampus as well as the perirhinal, insular, and medial prefrontal cortices. This task is based on the tendency for rodents to explore novel objects over familiar ones. However, impairments in recognition memory lead to the inability to discriminate between the two objects. In the present study, we found a significant effect of genotype on the discrimination of novel vs. familiar objects with 2N mice spending more time with novel objects but Ts65Dn mice spending similar amounts of time with both novel and familiar at ages 10 and 13 months. These findings are consistent with other reports in Ts65Dn mice.

In the cued and contextual fear conditioning task, cued (tone + light) fear conditioning is heavily dependent on the amygdala and contributions from the perirhinal and entorhinal cortices, and the thalamus. In contrast, contextual fear conditioning is predominantly dependent on the hippocampus with crucial inputs from the cerebellum. In the current study, we observed reductions in cued fear conditioning at 10 months, but not at 13 or 7 months of age. Previously, 6-month-old Ts65Dn along with other mouse models of DS demonstrated reduced freezing when reintroduced in the contextually conditioned chamber but demonstrate a normal freezing response to cued conditioning, suggesting intact amygdala functioning in these mice. It is not clear why our findings differ from our previous report of impaired freezing in context but not with the cue at 6 months of age; however, it may be due to the use of a single shock pairing, whereas 2 shock pairings were used in the present study. Perhaps this stronger conditioning overcame the contextual conditioning deficit.

In the Y-maze, a test of working memory, wild-type mice spontaneously alternate between the arms of the maze. We found that while Ts65Dn mice were not impaired at any age, spontaneous alterations non-significantly decreased by 10 months of age and remained so at age 13 months. This finding is in line with previously reported observations of Ts65Dn mice between 9 and 12 months of age, as well as another report on Ts65Dn mice at age 11 months.

### 4.4. Age and genotype

In the present study, we examined the impacts of age and genotype, comparing the Ts65Dn mouse model of DS with its 2N (euploid) control. After age 10 months we saw reductions in both genotypes for brain Aβ1-40 and total Tau and increases in soluble pTauS396 as well as plasma NfL after 8.5 months of age. At this same age in the Ts65Dn mouse the deficits in NOR were more marked and continued so in older mice. In Ts65Dn mice only, between 13 and 16 months we saw a marked increase in insoluble pTauS396. The changes in the 10–16-month age range may well define a period during which increased DS-AD-related pathogenic events were registered. Other DS-AD-related changes were previously reported in Ts65Dn mice. Reductions in locus coeruleus neurons and cholinergic neurons are evident by 6- and ~ 20 months of age, respectively. Marked dysregulation of the endosomal pathway and neurotrophin signaling is present by age 16 months (Chen et al., 2021). These data have implications for planning future experiments aimed at preventing disease-related phenotypes and biomarkers. Interventions should be planned to address specific manifestations using treatments and treatment durations adequate to engage targets to prevent the emergence of phenotypes.

## Data availability statement

The raw data supporting the conclusions of this article will be made available by the authors, without undue reservation.

## Ethics statement

The animal study was reviewed and approved by the Scripps Research Institution’s Animal Care and Use Committee and University of California San Diego (UCSD) Institutional Animal Care and Use Committees.

## Author contributions

EF, MV, CB, CM, RM, and VE conducted all biochemical experiments. AB performed all animal experiments. AR performed all behavior testing and initial analysis of behavioral results. CO performed all statistical analyses and drafted the manuscript. WM, AP, and MK-V conceived and designed the experiments. All authors read the final manuscript and participated in data analysis and interpretation and contributed to the manuscript revision, read, and approved the submitted version.

## Funding

This study was funded by a contract with the University of California San Diego from AC Immune.

## Conflict of interest

EF, MV, CB, CM, RM, VE, AP, and MK-V are all employees of AC Immune who funded this research. No products of AC Immune were included in this manuscript since only vehicle-treated mice were analyzed. WM serves as a consultant to AC Immune and Acta Pharmaceuticals. WM has grants or contracts from NIH (AG078241, AG070154, AG067035, AG077148, and P01NA092525), Larry L. Hillblom Foundation (2019-A-006-NET), Michael J Fox Foundation (ASAP-020566 and MJFF-020705), Ono Pharma Foundation UCSD 2019–0742, BioSplice Inc., ACI Immune SA, Alzheimer Association DSAD-15-363,207, Cure Alzheimer’s Fund, and Annovis Bio. WM has Royalties or licenses from Curasen Inc. licensed by Stanford (no payment to WM). WM received consulting fees from Samumed, AC Immune SA and Pfizer Inc. WM received support for attending meetings and/or travel from the National Down Syndrome Society, Sanford Health, AC Immune, U Michigan, ASAP Meeting, Sanford Health, Burke-Blythedale Meeting (New York), and ADRD and COBRE meetings (University of Nebraska, Medical Center). WM has patents planned, issued, or pending for the use of gamma-secretase modulators held by UCSD and Harvard. WM received stock or stock options from Annovis Bio (as payment), Cortexyme (as payment), and Alzheon (not as payment). WM received study drug and gift funding to the lab from Annovis Bio.

The remaining authors declare that the research was conducted in the absence of any commercial or financial relationships that could be construed as a potential conflict of interest.

## Publisher’s note

All claims expressed in this article are solely those of the authors and do not necessarily represent those of their affiliated organizations, or those of the publisher, the editors and the reviewers. Any product that may be evaluated in this article, or claim that may be made by its manufacturer, is not guaranteed or endorsed by the publisher.
