# Supplementary material for: Modeling Alzheimer’s disease related phenotypes in the Ts65Dn mouse: impact of age on Aβ, Tau, pTau, NfL, and behavior
Source: Front Neurosci. 2023 Jun 28;17:1202208. doi: 10.3389/fnins.2023.1202208 (PMC10336548; doi:10.3389/fnins.2023.1202208)
Supplement: Supplementary file 1 [file Presentation_1.PPTX]

## Slide 1
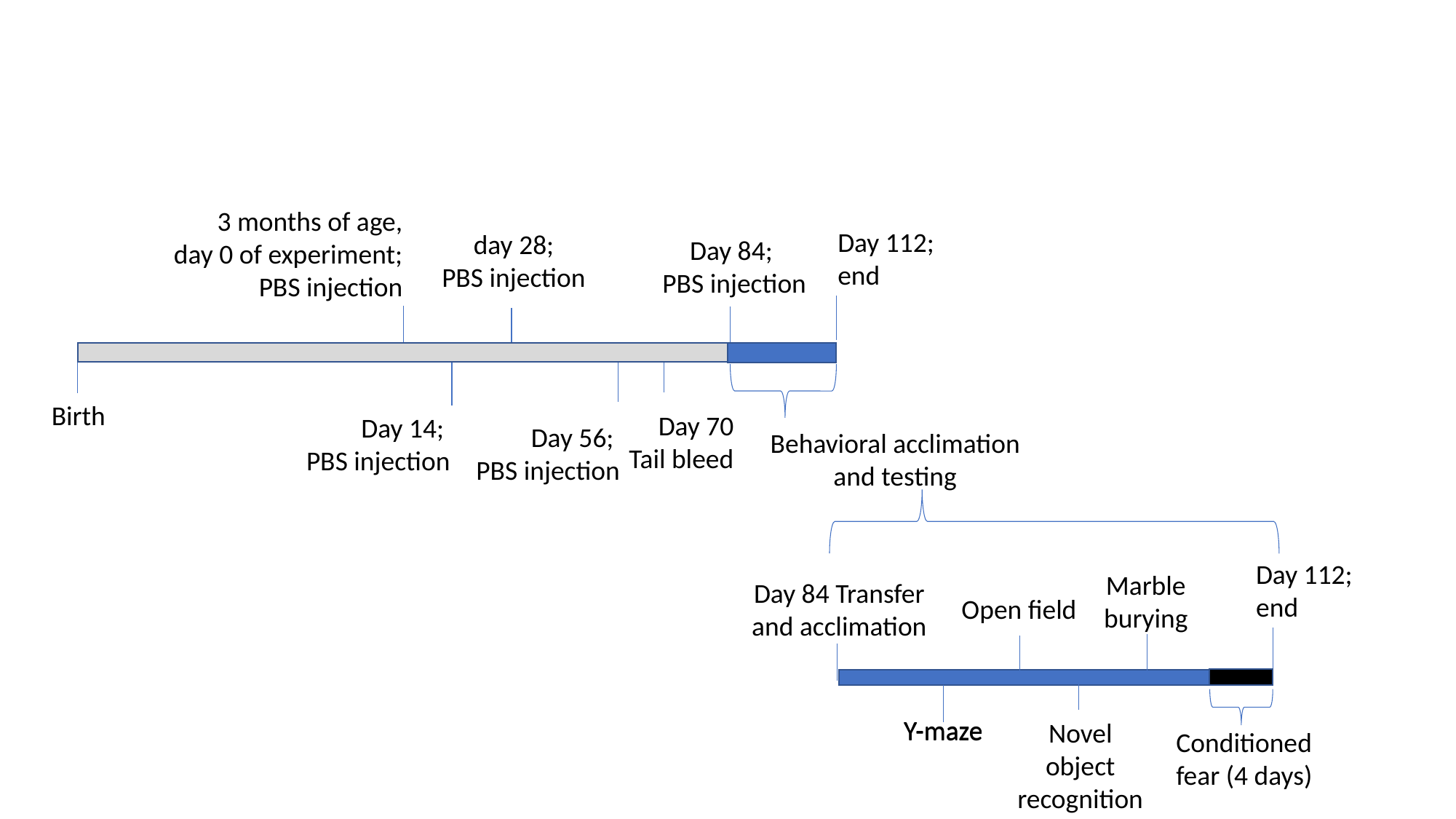

3 months of age,
 day 0 of experiment;
PBS injection
Day 112;
end
day 28;
PBS injection
Day 84;
PBS injection
Day 56;
PBS injection
Day 14;
PBS injection
Birth
Behavioral acclimation and testing
Day 70
Tail bleed
Day 112;
end
Marble burying
Day 84 Transfer and acclimation
Open field
Y-maze
Y-maze
Novel object recognition
Conditioned fear (4 days)
