# Supplementary figures and images for: Modeling Alzheimer’s disease related phenotypes in the Ts65Dn mouse: impact of age on Aβ, Tau, pTau, NfL, and behavior
Source: Front Neurosci. 2023 Jun 28;17:1202208. doi: 10.3389/fnins.2023.1202208 (PMC10336548; doi:10.3389/fnins.2023.1202208)

## Slide 1
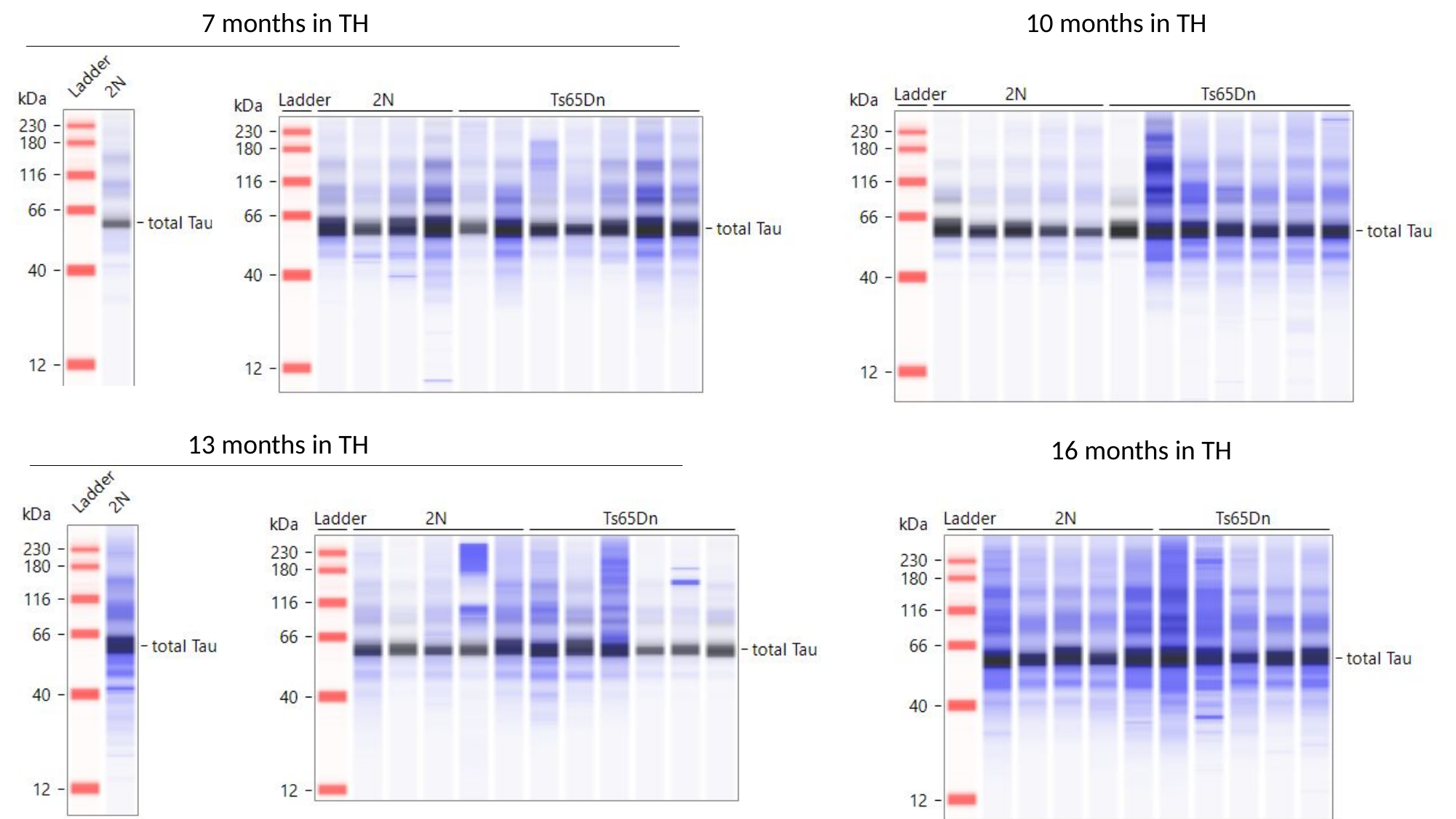

7 months in TH
10 months in TH
13 months in TH
16 months in TH

Supplement: Supplementary file 2 [file Presentation_2.pptx]

## Slide 1
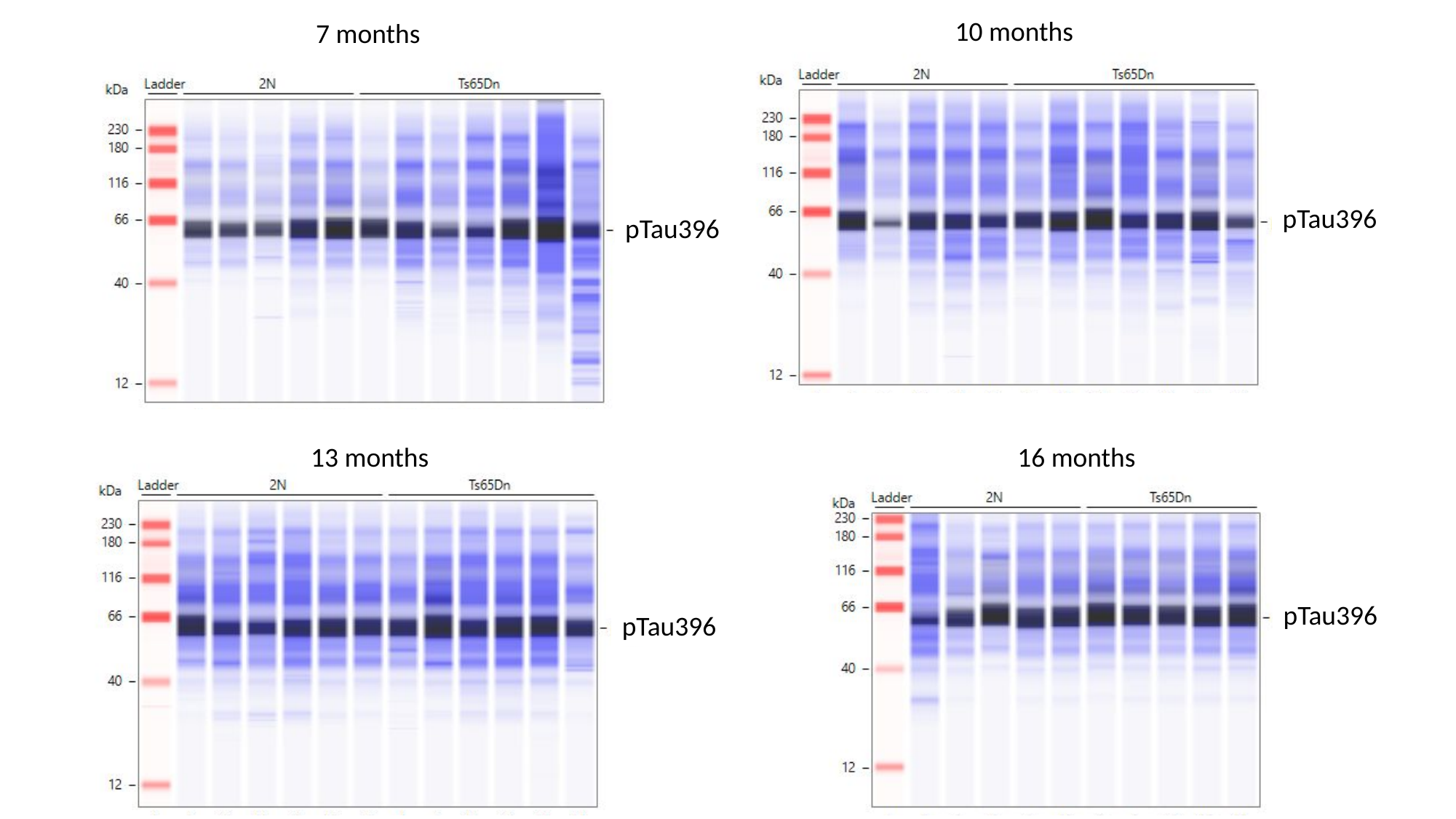

10 months
pTau396
7 months
pTau396
13 months
16 months
pTau396
pTau396

Supplement: Supplementary file 3 [file Presentation_3.PPTX]

## Slide 1
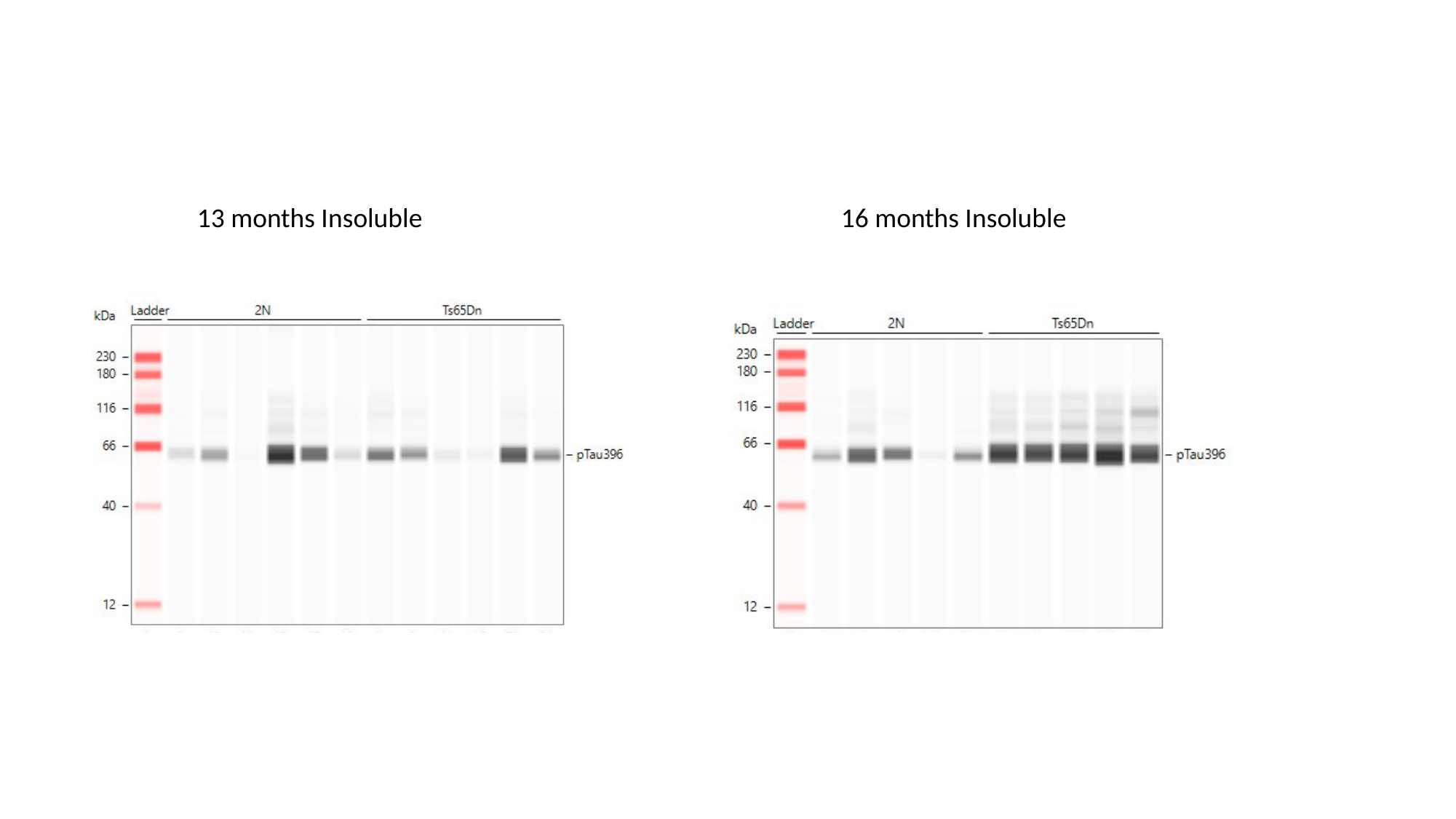

13 months Insoluble
16 months Insoluble

Supplement: Supplementary file 4 [file Presentation_4.PPTX]
