# Supplementary material for: Modeling Alzheimer’s disease related phenotypes in the Ts65Dn mouse: impact of age on Aβ, Tau, pTau, NfL, and behavior
Source: Front Neurosci. 2023 Jun 28;17:1202208. doi: 10.3389/fnins.2023.1202208 (PMC10336548; doi:10.3389/fnins.2023.1202208)
Supplement: Supplementary file 5 [file Data_Sheet_1.docx]

Supplementary Figure 1: Timeline of experimental procedures using the mice that were 3 months at the start of the experiment and 7 months at the time of sacrifice. The same protocol was used for mice but starting at ages 6-, 9-, and 12- months of age and ending at 10-, 13- and 16-months of age, respectively.

Supplementary Figure 2: Representative Western blot images of total Tau as measured by WB in total brain homogenates of 2N and TS65Dn mice at different ages. Total-Tau was detected using BD biosciences 556319 (dilution 1:30) followed by HRP-conjugated secondary antibody (ProteinSimple). The quantification of protein levels is presented in Figure 2C.

Supplementary Figure 3: Representative Western blot images of pTau S396 protein level in total brain homogenates of 2N and TS65Dn mice at different ages measured. The quantification of protein levels is presented in Figure 2E.

Supplementary Figure 4. Representative Western blot images of pTau S396 protein level in Sarkosyl-insoluble fraction of the 13- and 16-month-old cohort. The quantification of protein levels is presented in Figure 2F.
